# Understanding risk with FOTRES?

**DOI:** 10.1007/s43681-022-00223-y

**Published:** 2022-10-06

**Authors:** Tim Räz

**Affiliations:** Institute of Philosophy, University of Bern, Länggassstrasse 49a, 3012 Bern, Switzerland

**Keywords:** Recidivism risk assessment, Machine learning, COMPAS, FOTRES, Understanding, Fairnessy

## Abstract

The present paper examines the recidivism risk assessment instrument FOTRES, addressing the questions whether FOTRES provides us with an adequate understanding of risk, whether we actually understand FOTRES itself, and whether FOTRES is fair. The evaluation of FOTRES uses the criteria of empirical accuracy, representational accuracy, domain of validity, intelligibility, and fairness. This evaluation is compared to that of COMPAS, a different, much-discussed risk assessment instrument. The paper argues that FOTRES performs poorly in comparison to COMPAS with respect to some of the criteria, and that both FOTRES and COMPAS do not show a satisfactory performance with respect to other criteria.

## Introduction

The use of algorithms in domains of high social relevance has been much debated in recent years. In particular, the case of COMPAS, a recidivism risk assessment instrument widely used in the USA, has sparked a lively discussion. In 2016, ProPublica [2] charged COMPAS with being biased against black people. The ProPublica investigation prompted a surge of research in computer science on the fairness of machine learning algorithms (fair-ML).^1^ Unfortunately, other risk assessment instruments, in particular outside the USA, have received less attention. The present paper takes a closer look at one such instrument, FOTRES, which is currently used in Switzerland and Germany. I will address the question whether FOTRES provides us with an adequate understanding of risk, and whether we actually understand FOTRES itself. I will also discuss the fairness of this instrument.

Risk assessment instruments take a scientific approach to risk. They are supposed to reliably predict the risk that an offender will reoffend in the future. Therefore, risk assessment instruments should satisfy scientific standards; this is the methodological starting point of the present paper. The paper draws on an established body of work from the philosophy of science and applies it in the context of recidivism risk assessment. It thus falls squarely into the category of *socially relevant philosophy of science* [16].

The paper yields three main outcomes. First, it introduces a novel set of criteria for evaluating risk assessment instruments, based on the debate on understanding in philosophy of science and on the debate on fair-ML. Understanding encompasses known criteria like (empirical) accuracy, but also intelligibility. Second, it establishes that FOTRES performs poorly in comparison to COMPAS with respect to some of these criteria. Third, it establishes that both FOTRES and COMPAS do not show a satisfactory performance with respect to other criteria.

I will first provide some background on recidivism risk assessment instruments and introduce both FOTRES and COMPAS (Sect. 2). After methodological stagesetting (Sect. 3), I will introduce four criteria of scientific understanding: empirical accuracy (Sect. 4), representational accuracy (Sect. 5), domain of validity (Sect. 6) and intelligibility (Sect. 7), drawing on work from philosophy of science. I will also introduce algorithmic fairness (Sect. 8). FOTRES and COMPAS will be evaluated with respect to these criteria, and the results will be compared.^2^ Finally, I will summarize the evaluation and discuss its ramifications (Sect. 9).

## Risk assessment instruments

### Basics of recidivism risk assessment

One of the main purposes of recidivism risk assessment,^3^ is to provide information about an individual that has committed an offense, specifically, the risk that this individual commits a further offense in the future. There is wide variation with respect to the kinds of individuals and offenses taken into account, and with respect to the kind of information provided about the risk of reoffending. In the present paper, I will focus on the predictive role of risk assessment tools; I will not discuss the usefulness of these tools for purposes such as guiding treatment of mentally ill offenders.

It is common to distinguish different kinds of risk assessment [13]. Unstructured professional risk assessment means that domain experts judge the risk of reoffending on the basis of their expertise without formal guidance. Then, there are two kinds of structured risk assessment. Actuarial risk assessment uses items thought to be strongly associated with recidivism to predict a probability of reoffending, a recidivism score. Structured clinical instruments are used to predict risk categories, e.g., low, medium, or high risk, which should not be interpreted as a probability of reoffending; assessors use the risk category to reach a decision. Both COMPAS and FOTRES are structured risk assessment instruments. COMPAS is an instrument in the actuarial tradition, while FOTRES is a structured professional instrument.

There are many ways of evaluating risk assessment instruments [15, 48, 50]; here, some important metrics will be mentioned. First, the predictive validity measures whether a risk assessment tool predicts risk accurately. Second, inter-rater reliability is the degree to which different assessors agree on the input items for one and the same offender. The kinds of risk assessment instruments mentioned above differ with respect to these metrics. First, according to [13, p. 3], structured risk assessment is more reliable and more accurate than unstructured risk assessment. Second, unstructured professional judgement was found to be least accurate, structured professional judgement was intermediate, and actuarial assessment ranked highest among structured instruments in a meta-analysis of risk assessment for sex offenders [22].^4^

Further important properties of risk assessment instruments are the kinds of factors they use in their prediction. Static factors obtain once and for all, while dynamic factors can vary over time. An example of a static factor is criminal history, an example of a dynamic factor is current substance use. Some risk assessment tools are proprietary, i.e., privately owned, which often means that the company providing risk assessment does not disclose some aspects of the risk assessment tool, such as its internal structure, to the public and the agencies using the instruments. Both COMPAS and FOTRES are proprietary.^5^

### Introduction to FOTRES

FOTRES (“Forensic Operationalized Therapy/Risk Evaluation System”) is a recidivism risk assessment tool, but also offers the possibility of assessing treatment progress and interventions.^6^ It was first developed in Switzerland in the 1990s. FOTRES is a structured professional instrument and provides outputs in the form of risk categories for reoffending with respect to a particular offense, but without a specific time interval within which reoffending is to be expected. FOTRES was originally developed to assess and manage recidivism risk of violent and sex offenders.

FOTRES estimates the recidivism risk for a specific offense, the so-called target offense. Target offenses are specified on a fine-grained level, e.g., homicide, instead of estimating violent recidivism. FOTRES has two levels. The first level, risk–need assessment (RNA), estimates the recidivism risk and the treatability of an offender. The second level, risk management (RM), describes treatment progress and changes in recidivism risk due to the treatment. RNA is assessed once (at the time of the first offense, before treatment); RM is assessed periodically when the current recidivism risk needs to be known. In the present paper, the focus is on the RNA module. The RNA module comprises three kinds of input variables, which are summarily described here:

1.) *Baseline risk:* These 110 factors are potentially responsible for an offense. There are two types of baseline risk factors. 1.a.) *Risk profile:* These are 97 personal risk characteristics. Factors are grouped into “attribute groups”; to give an example, the group “problems with dominance” comprises the factors “desire to control”, “striving for dominance”, “ignoring the desires of others”. 1.b.) *Relevance of risk profile:* These 13 factors quantify “ how relevant the established risk profile is in explaining the offense mechanism” (Ibid, p. 244).

2.) *Plausibility of baseline risk:* These 26 factors serve as a check on the baseline risk score. They come in two types: 2.a.) *pattern of the offense*, and 2.b.) *offense-related personality dispositions*. Scoring these factors is optional; the result can be used to check the validity of baseline risk.

3.) *Baseline treatability:* These 26 factors measure the potential of the offender to change behavior through therapy. They come in two types: 3.a.) *perspectives for treatment*, and 3.b.) *personal resources*.

A subset of the 175 input variables are selected based on the target offense and rated on a scale ranging from 0 to 4. The scale of the input variables has the following interpretation: 0 = the risk characteristic is not present, 1 = present to a low extent, 2 = moderate, 3 = high, 4 = very high. The rating process is complex.^7^ If all information has been collected, it takes 60 min to rate one offender according to Gonçalves et al. [19, p. 248].

The RNA module provides four outputs: one for 1.) baseline risk, two for 2.) plausibility of baseline risk—one for 2.a.) and one for 2.b.) –, and one for 3.) baseline treatability. The two plausibility scores are supposed to be compared to the baseline risk. There is no prescription or rule regarding how these scores are supposed to be related; however, differences could indicate a problem with either one of the scores. Scores for baseline (and current) *risk* are on a scale between 0 and 4 in .5 increments, with the following interpretations: 0–0.5 = very low risk for committing crimes in the domain of the assessed target offense, 1 = low risk, 1.5 = low to moderate, 2 = moderate, 2.5 = moderate to high, 3=high, 3.5 = high to very high, 4 = very high. FOTRES is a structured professional instrument, which means that it does not provide recidivism rates. However, depending on the output, FOTRES provides the user with a “specific interpretation of the recidivism risk and indications for treatment, as well as specific recommendations for treatment and its prospects” (Ibid. p. 249). Scores for baseline (and current) *treatability* are on a scale between 0 and 4, in .5 increments, with the following interpretation: 0 = no treatability; 0.5 = very low treatability,... 4 = very high treatability.

How are the outputs computed from the inputs? Concerning baseline risk, the factors from 1.a.), risk profile, and from 1.b.), relevance of risk profile, first produce two separate scores; these two scores are then combined to produce the total score of baseline risk. The same applies to the factors in baseline treatability, i.e., the factors in 3.a.) and 3.b.). What does it mean that the values of the input factors are “combined to produce” the outputs? According to Gonçalves et al., “[a]ll total scores are calculated automatically by the web application. The details of the algorithm are not shown to the user to avoid human errors and manipulation of the results” —we will discuss this below. This means that while FOTRES provides an algorithm for risk assessment—a mechanical procedure to compute outputs from inputs—the details of this algorithm are hidden.

FOTRES is mandatorily applied in the assessment of high-risk offenders in German-speaking cantons in Switzerland, and there are plans to extend this to French-speaking cantons, and eventually to all of Switzerland (Ibid., p. 250) in the context of the so-called “risk-oriented penalty enforcement” system (“Risikoorientierter Sanktionenvollzug”, ROS). According to Hahn [21], FOTRES is one of the most commonly used instruments in Switzerland. FOTRES is also used in other European countries; Rettenberger [40] reports that FOTRES is one of the most commonly used risk assessment tools for violent recidivism in Germany.

### Introduction to COMPAS

COMPAS (“Correctional Offender Management Profiles for Alternative Sanctions”) is an actuarial risk assessment tool.^8^ Its purposes include prediction of violent recidivism, general recidivism, and failure to appear, but it can also be used in rehabilitation and treatment. There are versions for young offenders (“Youth COMPAS”), for offenders after longer periods of incarceration (“Reentry COMPAS”), and for female offenders (“Women COMPAS”).

COMPAS was developed in the USA in the 1990s, taking LSI-R, then a state-of-the-art risk assessment tool, as a conceptual starting point. COMPAS uses static as well as dynamic risk factors. COMPAS is proprietary and owned by the company Equivant.^9^ It is one of the most used risk assessment instruments in the USA.^10^ While COMPAS comes in a variety of versions and with different functionalities, here the focus is on “Core COMPAS”, which comprises two models, one for predicting violent recidivism and the other for predicting general recidivism. The two models take information about defendants as input. The input is based on 137 questions which are put to defendants after arrest.^11^ Questions include items such as: Have your parents ever been sent to jail or prison? and: How many of your friends/acquaintances take illegal drugs? The models output decile risk scores, numbers between 1 and 10. Scores 1–4 are labeled “Low”, 5–7 are labeled “Medium”, and 8–10 are labeled “High” by COMPAS. The predicted risk is that of reoffending within 2 years of the first arrest (time of assessment). According to Desmarais et al. [13], it takes 10–60 min to administer COMPAS.^12^

Concerning the question of how the risk scores of the two models are computed, the details are not public; however, a general description is available [8, 9]. The model for general recidivism risk is a linear model derived from a LASSO regression model, trained on historical data from 2002. The model for violent recidivism is a regression model developed in 2006. From the description of the developers of COMPAS, we can see that this instrument was developed using standard methods from machine learning and statistics, cf. Hastie et al. [23].

COMPAS is not only widely used, it is also widely discussed. In 2016, ProPublica published a critical evaluation of COMPAS.^13^ Through a public records request, ProPublica obtained data from Boward County, Florida, containing the records of over 7000 people arrested in that county. These records included the risk scores assigned by COMPAS to these defendants at the time of their arrest. ProPublica added the information whether the defendants actually were charged with new crimes for a period of two years after their first arrest (and after being scored by COMPAS).

Based on these data, ProPublica evaluated COMPAS along several dimensions. They found: first, COMPAS correctly predicted offenders’ recidivism 61% of the time, and violent recidivism 20% of the time. Second, COMPAS predicted black and white offenders’ recidivism with approximately the same accuracy (63% for black offenders, 59% for white offenders). Third, black defendants were found to have an (unfavorable) higher false positive rate (45%) than white defendants (23%). Qualitatively, the same is true for violent recidivism. Fourth, white defendants were found to have a (favorable) higher false negative rate (48%) than black defendants (28%). Qualitatively, the same is true for violent recidivism. All of these relationships were shown to be robust even when controlling for factors such as prior crimes, future recidivism, age and gender. In reaction to the ProPublica evaluation, Northpointe, the company owning COMPAS at that time, as well as other researchers [17], criticized the methodology employed by Angwin et al. [2], with a focus on the adequacy of the measure of fairness used in the study.

## Methodology

The evaluation in this paper draws on the concept of scientific understanding as discussed in philosophy of science.^14^ To illustrate the importance of understanding, take climate modeling as an example. When we study climate phenomena, such as climate change, we build climate models not only to get predictions, but also to gain a better understanding of how climate change comes about, so that we know how it can be mitigated, and so on. When we use models to understand something about the world, we also want to understand the models themselves. After all, if we use a model to gain knowledge about the world, that is, if we use it as an instrument of science, we should also understand how the instrument itself works, in order to make sure that it does what it is supposed to do; this point will be elaborated in Sect. 5 below.

*Prima facie*, it could be thought that structured risk assessment is very different from climate modeling. However, on closer inspection, structured risk assessment instruments raise similar issues. These instruments should predict whether an individual that has committed an offense will reoffend in the future with a certain degree of accuracy. However, just as in the case of climate models, accurate predictions are not enough. We also want to understand how such an instrument produces predictions. For example, the factors used in the prediction, and the model structure, should reflect our current theoretical knowledge about recidivism risk. Even more importantly, those subjected to risk assessment arguably have a right to understand how a prediction affecting them came about; see, e.g., Vredenburgh [56]. To make sure that these desiderata are met, it is necessary to have access to the inner workings of risk assessment instruments, and to gain a certain degree of understanding of how they work. I will detail these points in the discussion of the criteria of understanding below.

Philosophers of science have proposed and discussed criteria for scientific understanding. Here I will use a specific set of criteria, proposed in Jebeile et al. [27] for the context of climate modeling, and adapt it for the purpose of evaluating risk assessment instruments.^15^ The four criteria for understanding are: 1. empirical accuracy, the degree to which a model makes accurate predictions; 2. representational accuracy, the degree to which the structure of the model captures the structure of the target system to be modeled; 3. domain of validity, the degree to which we understand the purpose of the instrument and the population to which the instrument is applied; 4. intelligibility, the degree to which different (groups of) agents have access to, and can grasp, aspects of a model. These four criteria, together with fairness, provide a novel way of evaluating risk assessment instruments from a scientific perspective. The field of risk assessment has its own criteria for evaluating risk assessment instruments, some of which were mentioned in the previous section. I will explain how these standard criteria fit into the present framework along the way.

The goal of the present paper is to evaluate structured risk assessment with scientific criteria. I will not discuss the question whether it is appropriate to take a scientific approach to risk assessment in detail. However, it seems clear that the scientific adequacy of a risk assessment tool has wider social and legal ramifications. For one, methods that are legitimized via science should also be evaluated according to scientific standards.^16^ What is more, in the Swiss context, where FOTRES is used, it is a legal requirement that tools contributing to forensic reports comply with scientific standards [57]. Thus, scientific criteria bear directly on legal and social issues. This will be elaborated below.

Finally, it is important to state clearly what the following evaluation does and does not tell us about COMPAS. COMPAS is used a proxy for the state of the art: COMPAS is widely used, it has been empirically evaluated by different, independent researchers, and it has been intensely scrutinized, in particular with respect to the dimensions of accuracy and fairness. Its virtues and drawbacks are well-known. By using COMPAS as a benchmark, I am not advocating its use. COMPAS, and the state of the art, have severe shortcomings. Despite this, COMPAS can help us gauge the relative merits of FOTRES: If the comparison of FOTRES with COMPAS shows that FOTRES performs significantly worse than COMPAS, this shows that FOTRES is flawed *in comparison to an actually existing alternative*, however flawed, in an absolute sense, this alternative may be. The reverse of this conditional is that if COMPAS is not a viable risk assessment instrument, then, *a fortiori*, FOTRES is not viable either.

## Empirical accuracy

### Description

Empirical accuracy, the first criterion, is the degree to which model outputs match observational data. Empirical accuracy can be determined independently of the inner structure of a model. It depends on an appropriate relation between the model and the world. A certain degree of empirical accuracy is a necessary condition if we want to understand with a model. An instrument that is supposed to predict the risk that a violent offender will reoffend should be able to provide consistent predictions that match actual violent recidivism, at least to a certain degree.

I will consider four aspects of empirical accuracy. The first two aspects are well known in recidivism risk assessment.^17^ First, reliability is the degree to which ratings (inputs for the instruments) are consistent. In particular, inter-rater reliability measures the degree to which different raters agree in their assessment of the same individual and thus arrive at the same ratings, which is necessary to arrive at consistent predictions.^18^ Second, predictive validity is the degree to which an instrument makes accurate predictions.^19^ Third, I will consider reevaluation practices. Reliability and predictive validity are always evaluated with respect to a particular model. If a model is modified after evaluation, reliability and predictive validity can be degraded, which makes it necessary to reevaluate the model. Fourth, I will examine whether the kind of output is standardized and easily comparable across instruments.

### Empirical accuracy of FOTRES

First, considering inter-rater reliability: In a recent discussion of FOTRES [20], the authors point out that there is just one small independent study investigating the inter-rater reliability of FOTRES [29]. Keller et al. found that the inter-rater reliability of FOTRES is in the low or medium range. Inter-rater reliability was also investigated by Rossegger et al. [42], who conducted a pilot study and found that inter-rater reliability for several tools, including FOTRES, was “excellent”. However, Rossegger et al. [42] is not an independent study; more on this below.

Second, considering predictive validity: The evidence for the predictive validity of FOTRES is quite thin. For one, no independent studies of the predictive validity of FOTRES have been carried out so far. Independent studies are important to avoid authorship bias. A recent survey [49] found that a significant authorship bias exists in evaluations of risk assessment tools: if the authors of an evaluation were also the designers of the risk assessment instrument under evaluation, the predictive validity of the risk assessment tool was found to be two times higher than in independent evaluations. Singh et al. [49] also write that conflicts of interest were routinely not reported in studies included in their survey.

According to Habermeyer et al. [20], only one study investigates the predictive validity of FOTRES, viz., Rossegger et al. [42]. Rossegger et al. found that FOTRES has a predictive validity, including AUC, that is in the same range as other, well-validated risk assessment instruments such as PCL-R and VRAG. In this sense, FOTRES has an acceptable predictive validity. However, Rossegger et al. is not an independent study, as Habermeyer et al. [20] note. At least three of the authors (Astrid Rossegger, Thomas Villmar, Jérôme Endrass) were involved in developing FOTRES.^20^ Furthermore, at least two of the authors (Astrid Rossegger, Jérôme Endrass) have co-authored three or more papers with Frank Urbaniok, the main developer of FOTRES, prior to 2011. Despite these facts, these authors of Rossegger et al. [42] did not disclose their involvement in the creation of FOTRES and their scientific collaboration with Frank Urbaniok, which constitute a conflict of interest, even though the *International Journal of Offender Therapy and Comparative Criminology*, where Rossegger et al. [42] is published, requires authors to disclose conflicts of interest. The results on authorship bias reported in [49] suggest that, as a consequence, the predictive validity of FOTRES found by Rossegger et al. is likely to be optimistic.^21^

Third, considering re-evaluation practices: several versions of FOTRES have been used so far. The description given in section 2.2 concerns FOTRES 3.0, the current version.^22^ According to Gonçalves et al. [19, pp. 246], various aspect of FOTRES have changed between versions: The overall structure has been improved, the scales and the calculation of some scales have changed, and there were also terminological and conceptual changes. Furthermore, FOTRES is “constantly being updated.” (Ibid., p. 246). Because FOTRES is proprietary, these changes cannot be tracked, and it is not possible to say how the differences between versions affect predictions. This lacuna could be overcome, to a certain extent, by re-evaluating the predictive validity and reliability of new versions. This, however, has not happened. The only evaluation of the predictive validity of FOTRES [42] concerns FOTRES 2.0. This means that the predictive validity of the currently used version, FOTRES 3.0, is not known.

Fourth, considering the prediction scale: FOTRES is a structured professional instrument and provides predictions in the form of eight risk (and treatability) categories, on a scale from “very low” to “very high” risk (or treatability). FOTRES does not provide a probability of reoffending. Risk categories, however, have some well-known problems. One is that there is no “well-subscribed method to create the categories” [52]. A second problem is that risk categories make it hard to compare different risk assessment tools. Arguably, probabilities also have drawbacks, e.g., by creating a spurious sense of precision. However, they make it easier to compare and assess risk assessment tools, and to improve scores. For example, Rossegger et al. [42] note that accuracy as measured by AUC is not the only relevant measure of a tool’s predictive accuracy; it is also desirable to have a calibrated score.^23^ Because FOTRES does not provide scores, calibration of the score cannot be calculated or enforced, as Rossegger et al. [42] acknowledge.

### Empirical accuracy of COMPAS

First, there is no independent study of the inter-rater-reliability of COMPAS according to Brennan and Dieterich [8], but an independent study of test-retest-reliability, which reports that COMPAS performs well with respect to this metric. Second, the predictive validity of COMPAS has been validated by independent groups from multiple geographic areas, also with a satisfactory performance in terms of, e.g., AUC. Third, COMPAS is periodically re-normed, re-validated, and calibrated for “large client agencies” [8, p. 52] on new samples. Fourth, COMPAS provides risk scores—it is an actuarial instrument—and the score is calibrated.

In sum, the performance of FOTRES is either unsatisfactory or has not been independently verified with respect to all aspects. COMPAS fares better than FOTRES with respect to at least three of the four aspects of empirical accuracy.

## Representational accuracy

### Description

The second criterion for understanding is representational accuracy, the degree to which the structure of a model captures relevant features of the target system we want to capture. In the case of risk assessment instruments, this means that the factors that serve as inputs of the model should be relevant to risk, comprehensive, and grounded in relevant theories from (forensic) psychology and psychiatry. It also means that a risk assessment instrument should adequately capture the interactions between these factors.

While representational accuracy and empirical accuracy clearly depend on each other, representational accuracy does not reduce to empirical accuracy, and should be checked independently, because we do not only want a model to make the right predictions, we also want it to make the right predictions for the right reasons. This is important for several reasons. First, it may be necessary to understand how a prediction came about in order to justify it. Second, if we are confident that our model adequately captures relevant aspects of the target system, this may lead to more stable predictive performance even if new data is somewhat dissimilar to the data on which an instrument was originally tested.^24^ Evaluating representational accuracy is more qualitative than empirical accuracy in the case of recidivism risk assessment.

A fundamental problem with evaluating representational accuracy is that the internal structure of both FOTRES and COMPAS is not fully accessible because the instruments are proprietary. I will discuss those aspects of the representational accuracy of risk assessment instruments to which we have access. First, I will rely on information about the type of instrument we are dealing with. Second, I will discuss the of kind of input factors the instruments use, in particular the number of factors, and what is known about the use of static and dynamic factors. Third, I will consider the theoretical foundations of the instruments in psychology and psychiatry. Fourth, I will comment on the logic of the modular structure of the instruments.

### Representational accuracy of FOTRES

First, consider the type of instrument: FOTRES it is a “structured professional instrument”. Rossegger et al. [42] discuss properties of risk assessment tools they consider to be important and conducive to understanding risk. What is supposed to distinguish FOTRES from actuarial risk assessment tools is, among other things, the large number of factors it takes into account. Rossegger et al. write: “Unlike the reductionism of actuarial scales, clinical instruments attempt to assess the complexity of a case, which allows professionals in the field to better understand the offender and thus to better plan suitable interventions.” (Ibid., p. 720) *Prima facie*, this sounds plausible. However, there is no empirical evidence supporting the claim that the large number of factors leads to an increase in understanding. For one, as we have seen in the last section, FOTRES is by no means more empirically accurate than actuarial instruments. Also, there is empirical evidence that, generally speaking, structured professional instruments are not superior to actuarial instruments: A recent empirical study [22] examined the accuracy of recidivism instruments for sex offenders and found that when it comes to predictive validity, actuarial instruments perform better than structured professional instruments such as FOTRES.

Second consider the kind of input factors: Rossegger et al. claim that the inclusion of dynamic factors in FOTRES is beneficial, e.g., because they make it possible to document change. However, there is no empirical evidence proving this claim. Generally speaking, there is evidence that most of the predictive validity of structured instruments comes from static factors; see Taxman [52, pp. 273], and that instruments including dynamic factors could inflate risk in comparison to tools that only use static factors (Ibid.).

Third, consider the theoretical foundations: This aspect of FOTRES is discussed in Habermeyer et al. [20]. Habermeyer et al. note that there is a wealth of well-founded, empirical work on these factors in forensic psychology and psychiatry. The risk factors used in FOTRES are repeatedly dubbed as “risk-relevant personality traits” (“risikorelevante Persönlichkeitsmerkmale”) by its creators. According to Habermeyer et al., conceptualizing these factors as personality traits would make it necessary to base them on personality psychology, e.g., the *Big Five* model. However, the risk factors used in FOTRES lack such a theoretical foundation. Habermeyer et al. write: “The status of ‘risk-relevant personality traits’ of FOTRES with respect to pertinent personality traits in terms of scientifically accepted conceptual systems is completely unclear” (Ibid., p. 216, translated by the author). Similarly, Habermeyer et al. note that the risk factors in FOTRES deviate from international standards concerning mental disorders and diseases as laid out in the “Diagnostic and Statistical Manual of Mental Disorders” (DSM) and the “International Classification of Diseases” (ICD). FOTRES uses non-standard terminology with respect to diagnostic criteria. This, Habermeyer et al. note, leads to the creation of a new diagnostic system, which has not been empirically evaluated, and creates ambiguities and possibly confusion.^25^

Fourth, consider the modules of FOTRES (see Sect. 2.2). The risk–need assessment (RNA) module has three components: baseline risk, plausibility of baseline risk, and baseline treatability. The second component, plausibility of baseline risk, is optional and supposed to serve as a check of baseline risk. *Prima facie*, this may seem like a good idea. However, it raises the question as to the status of the factors included in this component. Are the plausibility factors, which are not included in the computation of baseline risk, actually predictive for recidivism risk? If the answer is yes, this raises the question why is it optional to include these predictive factors. If, however, the answer is no, then this raises the question as to how these factors can serve to check the baseline risk. Factors that do not contribute to risk cannot serve as a control for risk. Thus, either FOTRES does not use all relevant factors all the time, or it uses redundant factors as controls.

### Representational accuracy of COMPAS

COMPAS is a proprietary instrument, and while a general description of the methods used in creating COMPAS are known [8, 9], its structure is not publicly known. In this respect, FOTRES and COMPAS are on a par.

First, COMPAS is an actuarial instrument, and includes 137 items in its questionnaire. This means that its size of the item set is similar to that of FOTRES.^26^ Second, like FOTRES, COMPAS uses static as well as dynamic factors, and, like FOTRES, the creators of COMPAS claim that their instrument can be used for a multitude of purposes in a multitude of settings, including treatment. There appear to be few studies of the impact of COMPAS during treatment.^27^ Third, concerning the theoretical foundation of risk factors, Brennan and Dieterich [8, p. 57] write: “COMPAS assesses core variables from social learning theory, strain theory, control/ bonding theory, routine activities theory, social disorganization, and the General Theory of Crime”. However, it is impossible to judge whether the use of these theories is adequate without access to the structure of the model. Fourth, like FOTRES, COMPAS comes in many versions and has a modular structure, which can be adapted to the needs of different agencies and users. Brennan and Dieterich [8, p. 53] write that the scalability of COMPAS does not affect the basic risk models for general recidivism and violent recidivism, because the latter “are cordoned off and uninfluenced by such scale in/out selections”.

In sum, comparing the representational accuracy of FOTRES and COMPAS yields mixed results. On the one hand, COMPAS appears to rest on a sounder theoretical foundation than FOTRES, it uses state of the art methods, and more is known about its impact than in the case of FOTRES. On the other hand, the two risk assessment instruments share the problem that they are proprietary, and that it is therefore not possible to fully evaluate representational accuracy.

## Domain of validity

### Description

The third criterion of understanding is the degree to which we can delimit the domain of validity of a model. This means, first, that we have to determine whether the risk assessment tool is used for its intended purpose.^28^ For example, if a tool was built to assess general recidivism risk, it should not be used to predict violent recidivism risk. Also, the scope of concepts used to define offenses can change over time, e.g., if the definition of what constitutes a sexual offense is extended; and a tool needs to be adapted and re-evaluated accordingly. Second, understanding the domain of validity means that we have to know the structure of the population for which we intend to use it.^29^ This is important because the empirical accuracy of an instrument is closely related to the sample on which the instrument is evaluated. For example, if we evaluate the predictive validity of an instrument with respect to male offenders, this instrument might not provide us with accurate predictions with respect to females.

### Domain of validity of FOTRES

First, consider the properties FOTRES is supposed to predict. FOTRES was designed to predict reoffending for specific offenses. It was not designed to predict general recidivism, or violent recidivism. While the predictive validity of FOTRES has been investigated in Rossegger et al. [42], no empirical study of the predictive validity of specific offenses has been published. This means that FOTRES has never been empirically evaluated with respect to the predictions it is designed to make. One reason why the specific offenses have not been evaluated may be that it would be very time-consuming to separately evaluate all 29 target offenses available in FOTRES.

Second, concerning the structure of the population, we once more turn to Rossegger et al. [42]. The authors worked with a small sample of size $n=109$, which includes males from one Swiss penitentiary, Pöschwies, in the canton of Zürich. The sample includes only violent and sex offenders who were released from the maximum security unit. Foreigners with no permanent residence were excluded. This means that FOTRES has only been validated for a small sample of the population. There is no empirical evidence that it is appropriate to use FOTRES in application to non-violent and non-sex offenders, female offenders, or offenders that are foreign citizens. These problems notwithstanding, FOTRES is used for risk assessment in all German speaking cantons of Switzerland (in the context of ROS), with plans to extend its use to all of Switzerland, cf. [19, 21]. The offenses for which FOTRES is used in the context of ROS may not have a similar distribution as the evaluated sample.^30^ Applying FOTRES to female offenders and foreign citizens is highly problematic. Also, FOTRES was not re-evaluated before its use outside of Switzerland in Germany [40].^31^

### Domain of validity of COMPAS

Concerning kinds of predictions, COMPAS provides a general recidivism risk score as well as a violent recidivism risk score, among other predictions, and these scores have been independently validated. There are different versions of COMPAS, with different factors, for different segments of the population.^32^ Concerning population structure, COMPAS has been evaluated and tested with respect to different segments of the population, and there are different models for different social groups.^33^

In sum, while it is state of the art to re-evaluate a risk assessment tool for different domains, such as geographic regions, and check validity for different subgroups (gender, race), as witnessed by COMPAS, no comparable evaluation is available for FOTRES.

## Intelligibility

### Description

The fourth criterion is intelligibility, the degree to which an agent, or a group of agents, can grasp a model’s behavior, and its inner workings, and manipulate and reason about the model. Intelligibility is different from the other criteria of understanding in that it is agent-relative: it depends on an agent’s ability to engage with a model, instead of being only concerned with the relation between the model and the world.^34^

Intelligibility is necessary for understanding in addition to the other criteria because even if a model takes all relevant factors into account and processes them adequately, an agent may still not understand the inner workings of the model if the agent cannot grasp the inner workings or does not have access to it. We can ask about intelligibility with respect to the three other criteria of understanding. An agent who has access to the input–output profile of a model can thereby gain a certain degree of intelligibility. If an agent has access to the inner workings of a model, and can track how inputs are processed, the agent gains a different kind of intelligibility. Intelligibility may also be a legal requirement in some jurisdictions, including Switzerland.^35^

There are different reasons why intelligibility can be limited for certain (groups of) agents. Here I will distinguish two kinds of limiting factors: grasp and access. First, limited grasp. It can be cognitively too demanding to grasp a model if the model is too complex.^36^ Grasp can be limited if the model in question is only poorly understood by science at present, or too complex in principle. In the case of physical modeling, we may not (yet) know how to solve the underlying physical equations, or they can only be solved numerically, or via simulations, which, in turn, may be opaque.^37^ The same is true in the context of machine learning, where different kinds of models may lack what is called interpretability or explainability, that is, they are not (yet) understood or hard to interpret in principle.^38^ Second, intelligibility can also be limited by access. Some agents can be denied access to a model, or certain aspects of the model by *fiat*, even though the agents would be able to grasp aspects of the model if they had access.^39^

### Intelligibility of FOTRES

Consider the groups of agents that have an interest in understanding FOTRES. The first group are those subjected to FOTRES, i.e., the offenders. The second group are those using FOTRES, i.e., professionals in the criminal justice system. The third group are those ultimately responsible for the use of FOTRES, viz., policymakers, and the general public. The fourth group are domain experts and researchers, in particular in the field of risk assessment instruments. The fifth group are the developers of FOTRES.

None of the above groups has unrestricted access to the inner workings of FOTRES, except for its developers. FOTRES is a proprietary algorithm, it belongs to Profecta AG, a private company that owns the copyright for FOTRES.^40^ It is unclear whether intelligibility is also limited due to grasp. For all that is known, FOTRES is not a particularly complex algorithm, and it may be graspable in principle.^41^ The lack of access to the inner workings of FOTRES constitutes a serious limitation on the intelligibility of FOTRES.

Of course, there are reasons for denying access to the inner working of FOTRES. One of them is given by Gonçalves et al. [19, p. 249]: “[D]etails of the algorithm are not shown to the user to avoid human errors and manipulation of the results.” However, it is not clear that keeping the model private avoids this problem. For one, it is possible to tamper with a private model solely based on the input–output profile.^42^ And even if it were the case that keeping a model private during deployment is useful, this does not mean that it also makes sense to keep it private when not deployed. In particular, agents like researchers and the general public have an interest in access even if they are not directly subjected to the algorithm. The most important reason for keeping FOTRES private, it seems, is economic. Access to FOTRES is sold to judicial agencies in Switzerland and Germany.^43^

Turning to reasons why the lack of intelligibility of FOTRES is problematic, first, this lack makes it impossible for experts and independent researchers to fully evaluate the representational adequacy of the model (cf. Sect. 5). Denying access prevents criticism and ultimately stands in the way of improving the model through a scientific and public debate. Experts using FOTRES only have a partial understanding of the reasons why individuals classified by FOTRES are assigned to a certain risk category. Experts thus have to accept predictions as brute facts, and, as a consequence, have to guess how predictions came about, instead of understanding predictions.

Second, keeping FOTRES private prevents offenders subjected to risk assessment from grasping why they are assigned to a particular risk category. Providing offenders with the input values that produced their classification in a certain risk category constitutes a partial reason for this classification at best. However, this is not sufficient, because the way in which the input is processed, which is determined by the inner working of FOTRES, is also relevant for the classification. Thus, denying access to FOTRES means denying offenders access to the reason why they are classified in a certain way, and, as a possible consequence, the reason why they are treated in a certain way. Vuille [57] argues that FOTRES is in violation of Swiss law for this very reason.^44^

### Intelligibility of COMPAS

Turning to the comparison between FOTRES and COMPAS, we find that the situation with COMPAS is similar. COMPAS is a proprietary instrument, and its internal structure is not fully known. Consequently, we face the same difficulties with respect to access to its internal structure. On the other hand, more is known about the empirical accuracy of COMPAS, which contributes to a higher degree of intelligibility, because for this instrument, we have a higher confidence that it does what it is supposed to do.^45^

## Fairnesss

### Description

Fairness is an issue of predictive modeling separate from understanding, but closely related to it: Fairness is related to empirical accuracy in that some fairness measures depend on the degree to which a predictor has the same accuracy across different groups. Also, if our understanding of the domain of validity is limited, this may lead to an unfair treatment of different groups, which, in turn, may be detected by measuring fairness. The issue of fairness has received increasing attention in recent years, prompted by the debate on COMPAS.^46^ In discussing the fairness of a particular model, we need to address several questions.

First, I will restrict attention to measures of group fairness that have been extensively discussed in the context of risk assessment instruments.^47^ Roughly speaking, group fairness is supposed to make sure that socially relevant groups are not treated differently without reason. It is usual to distinguish three main kinds of group fairness: independence, sufficiency, and separation. Independence requires that the prediction is statistically independent of group membership; sufficiency requires that the true outcome is conditionally independent of group membership given the prediction; separation requires that the prediction is conditionally independent of group membership given the true outcome.^48^

Second, the possibility of evaluating particular measures: measures of group fairness require that a model’s predictions satisfy certain statistical conditions with respect to socially relevant groups, such as gender, race, etc.. Measures of group fairness are formulated in terms of probabilities of three random variables, the prediction *R*, the “ground truth” (true outcome) *Y*, and the sensitive characteristic or group membership *A*. Thus, what is needed for evaluating these measures is historical data, with true outcomes (ground truth) and group memberships, for which the model can generate predictions.

Third, the performance in the evaluation of particular measures: This depends on the distribution of the historical data, but there are also some a priori (mathematical) constraints. Soon after the COMPAS debate started, it was discovered [10], that some group fairness measures can only be simultaneously satisfied under special circumstances as a matter of principle.^49^ The questions how to combine different measures of group fairness, and what partial fulfillment of these measures means, are still debated.

Fourth, can we modify the model such that it complies with these measures (to a certain extent)? Generally speaking, there are three strategies to enforce group fairness measures: By preprocessing the data, by enforcing fairness during training (i.e., during the construction of the model), and by post-processing, i.e., by modifying a model’s prediction so as to yield fairer outcomes; cf. Barocas et al. [3]. Whether it is possible to enforce fairness measures during training or in post-processing depends on the specifics of the model (e.g. whether it is possible to enforce fairness as a soft constraint during training, as in Kamishima et al. [28]) and on the kind of prediction generated by the model (e.g. whether the model provides scores).

### Fairnesss of FOTRES

First, can we evaluate the model with respect to group fairness measures? FOTRES is a clinical instrument, which assigns offenders to risk categories on a scale from “very low” to “very high” risk. This creates the problem that it is not clear how to operationalize these categories. What constitutes “very high” risk? If there is no principled way of relating predictions to the data, e.g. through probabilities (which correspond to reoffending rates), an evaluation is difficult because it is not clear how to compare predictions to ground truth, which is a problem for those fairness measures that are formulated using ground truth (i.e. variable *Y*). This difficulty can be circumvented to some extent by comparing the rates at which different groups are assigned to the different risk categories.^50^ Also, it is possible to use some risk category as a threshold, above which we interpret the category as “will reoffend”, and compare the distribution of different groups with respect to the resulting binary prediction and ground truth. In this sense, it is possible to evaluate the fairness of FOTRES with respect to the three main group fairness measures.

It should be noted that some measures that are related to fairness cannot be evaluated in the case of FOTRES in principle, due to the fact that FOTRES does not provide a risk *score* in the form of a probability of reoffending. Rossegger et al. [42] acknowledge that it would be desirable to evaluate their instrument with respect to calibration of the score, which is closely related to sufficiency. This, however, is not possible because calibration presupposes probabilistic predictions.^51^

Second, how does the model perform with respect to these measures? The answer to this question is unknown. We have just seen that it would in principle be possible to evaluate the fairness of FOTRES to a certain extent, but such an empirical evaluation has never been carried out. It would be desirable to have an independent evaluation with respect to fairness metrics, similar to an evaluation of empirical accuracy. One major obstacle to such an evaluation is a lack of empirical data. In Sect. 6, we have already seen that so far, the empirical accuracy FOTRES has only been evaluated on a small dataset, which is not representative of the population to which FOTRES is applied. One of the major practical problems for an evaluation of fairness is a lack of data on actual reoffending rates for some socially relevant groups. To give an example, it is practically impossible to evaluate the fairness of FOTRES with respect to a potential difference between Swiss and foreign citizens, because foreigners without permanent residence may be deported after having served their sentence, which means that following up on reoffending is presumably very hard, cf. Rossegger et al. [42]. Of course, the lack of data is not a problem of FOTRES in particular, but a problem for the evaluation of any risk assessment instrument with respect to fairness.

Third, can we modify the model such that it complies with fairness measures (to some extent)? It is hard to answer this question because the structure of the model, and the method of its construction, are not completely transparent. If FOTRES does not rely on a learning algorithm for its construction, as it appears to be the case (cf. Sect. 2.2), it is not possible to add fairness as a soft constraint during training. This means that the model would have to be modified by hand to make it comply with fairness measures, which may be a hard task. Also, FOTRES apparently does not rely on a risk score for creating the risk categories. This means that one important method of fairness post-processing, viz. modifying group-specific thresholds in order to satisfy fairness measures, cannot be applied to make FOTRES fairer.

### Fairnesss of COMPAS

As a result of the ProPublica evaluation, COMPAS has come under intense scrutiny, and has been repeatedly evaluated with respect to measures of group fairness. While COMPAS does not comply with balanced false negative and false positive rates (which corresponds to separation), it is a calibrated instrument. The key differences between COMPAS and FOTRES are that, first, COMPAS has been empirically evaluated with respect to different fairness measures, which has not happened in the case of FOTRES. Second, it may be harder to modify FOTRES to make it comply with fairness metrics in comparison to COMPAS, because FOTRES presumably does not rely on a learning algorithm, which means that, say, fairness regularization methods that are used during training (cf. [28] and others) cannot be applied to FOTRES, while this is possible for COMPAS.

## Summary and discussion

To recapitulate, FOTRES was evaluated with respect to four criteria of understanding: empirical accuracy, representational accuracy, domain of validity, intelligibility, and with respect to group fairness. The most important results of the evaluation, and the corresponding results for COMPAS, are summarized in table 1 (the first column contains abbreviations of the four criteria of understanding, and fairness).

**Table 1:** Evaluation summary. Abbreviations: EA = empirical accuracy, RA = representational accuracy, DV = domain of validity, I = intelligibility, F = fairness

Criteria and subcriteria of evaluation		FOTRES	COMPAS
EA	Adequate inter-rater reliability (shown by independent study)	No	Yes
	Adequate predictive validity (shown by independent study)	No	Yes
	Re-evaluations for new versions of instrument	No	Yes
	Instrument provides probability of reoffending	No	Yes
RA	High number of input items	Yes	Yes
	Use of dynamic factors	Yes	Yes
	Factors conform with scientific standards	No	?
DV	Adequate samples for domain of application	No	Yes
I	Access to internal structure, intelligibility	No	No
F	Evaluation w.r.t. group fairness measures	No	Yes

The outcome of the evaluation of FOTRES is unsatisfactory with respect to all criteria of evaluation. First, there is a lack of independent studies of the empirical accuracy of FOTRES. Second, FOTRES is insufficiently based on international scientific standards. Third, the nature of the instrument makes it hard to evaluate FOTRES and/or to compare FOTRES to other instruments, and it may be hard to modify FOTRES such that it complies with group fairness measures. Fourth, the proprietary nature of FOTRES makes it impossible to fully understand the instrument’s outputs, and to properly evaluate and criticize the inner structure of the instrument. All in all, from a scientific point of view, FOTRES is an unsatisfactory instrument.

What are appropriate measures to be taken in view of this situation? The question whether judicial agencies currently using FOTRES should stop using it cannot be addressed on the basis of a scientific evaluation alone. According to Vuille [57], the lack of scientific quality of FOTRES may have legal ramifications, because according to Swiss law, “expert opinions must rely on the latest scientific findings and experience” (Ibid., p. 28), which may not be given if individuals are assessed using FOTRES. It should be asked whether structured risk assessment should play a role in judicial systems in the first place; this fundamental decision cannot be based on science alone, it needs to be based on considerations of both science and values.^52^ If structured risk assessment should be used, it may be appropriate to replace FOTRES with a superior alternative.

If society decides that we want to use structured risk assessment instruments, we should answer two questions: What properties should a structured risk assessment instruments have? And: Are there instruments that have these properties? Providing a serious answer to the first question is beyond the scope of the present paper. However, the criteria used here could be part of an answer to this question, because an acceptable structured risk assessment tool should certainly be understandable and fair. Turning to the second question, if the criteria proposed here are taken as a starting point, then, in order to get a satisfactory degree of intelligibility, instruments like COMPAS that are not open source should be ruled out. There have been projects [1] to develop risk assessment instruments with a satisfactory degree of intelligibility—open source and with an interpretable structure—while also having a degree of accuracy comparable to other predictive models. Of course, such open source solutions still would have to be evaluated and adapted to the appropriate context, with the other criteria of understanding in mind.

## Footnotes

1 As witnessed, e.g., by the FAccT (“Fairness, Accountability, Transparency”) conference. See Barocas et al. [3] for an overview of fair-Ml.

2 See Vuille [57] for a critical comparison of FOTRES with HCR-20, a different risk assessment instrument, from a legal perspective.

3 See Singh et al. [48, 50] for recent surveys. Note that the terms risk assessment *instrument* r.a. *tool*, and r.a. *model* are used interchangeably in the present paper, although the terms carry different connotations in general.

4 According to Rettenberger et al. [41, p. 2] unstructured or intuitive criminal prognostics often have a level of accuracy close to random.

5 The fact that both instruments are proprietary means that the corresponding algorithms are not in the public domain, have not been published in a scientific journal, and reconstructing and disclosing these algorithms may constitute an infringement on copyright law; see, e.g., the licence conditions for FOTRES, https://www.fotres.ch/lizenzen/lizenzvertrag.html, accessed on June 16, 2021.

6 The official handbook for FOTRES 3.0, which is currently in use, is Urbaniok [54]; the description here follows Gonçalves et al. [19].

7 See Urbaniok [54, II.] for a full account of how to obtain a baseline risk score and Vuille [57, pp. 24] for a short description.

8 The account given here is based on [8].

9 Until 2017, COMPAS was maintained by Northpointe Inc.; cf. https://www.equivant.com/, accessed on Feb. 16, 2021.

10 See https://epic.org/ai/criminal-justice/, accessed on Feb. 17, 2021.

11 A sample questionnaire used for the core component of COMPAS used in Wisconsin was obtained during the investigation by Angwin et al. [2] and published online by Julia Angwin. Note that not all 137 items may need to be used for either risk score, cf. [26].

12 This variability in duration of administration may be due to the fact that different items are rated depending on the context.

13 [2]; see also the methodological companion [34].

14 See Baumberger et al. [4] for an introduction to understanding, Frigg and Hartmann [18, Sec. 3.4.] for an introduction to understanding with models, and de Regt [11] for an account of scientific understanding.

15 I use the evaluative criteria from Jebeile et al. [27], except for “physical consistency”, which has no counterpart in risk assessment. Note that the framework in Jebeile et al. [27] is similar to the framework proposed in Knüsel and Baumberger [30].

16 See, e.g., Imrey and Dawid [25], Berk [5] for commentary on methodological problems of risk assessment from a technical perspective, and Biddle [7] for a recent philosophical contribution. See also the references on fairness in Sect. 2.3 above.

17 See Singh et al. [50] and in particular Desmarais et al. [13] for a useful discussion of these concepts in the context of recidivism risk assessment.

18 Popular measures of inter-rater reliability are Kappa statistics. Kappa statistics, due to Cohen and Fleiss, measure the agreement of raters while correcting for baseline agreement or “agreement by chance”, cf. Landis and Koch [33].

19 Popular measures of predictive validity include AUC (area under curve), correlation coefficient, OR (odds ratio), and Somer’s *d*. To give an example, the AUC measures the probability that a randomly drawn individual that did in fact reoffend has a higher risk rating than a randomly drawn individual that did not reoffend; see Desmarais et al. [13], and Singh [47] for a methodological primer.

20 See the acknowledgements of [53].

21 This lack of transparency by some authors of Rossegger et al. [42], and the resulting problem with authorship bias, is also noted in Vuille [57].

22 According to www.fotres.ch, accessed on Feb. 2, 2021, the last version of FOTRES 2.0 has been discontinued in 2017, and the link to FOTRES 2.0 no longer works.

23 Calibration means that the predicted score corresponds to the actual risk level, e.g., if the prediction of reoffending for a certain subgroup is $20\%$, then, within that group, we should have an actual reoffending rate of $20\%$.

24 See Jebeile et al. [27] for more on the necessity of evaluating representational accuracy.

25 Note that the conceptual foundations of FOTRES have been critically discussed elsewhere in (forensic) psychiatry; see, e.g., Mokros et al. [37, p. 476] for criticism of the idea of “offense mechanism”, a key concept of FOTRES, and Nedopil and Müller [38, p. 363], who go as far as dubbing the belief in the possibilities of automated recidivism risk expressed in Urbaniok [54] a “profession of faith” (“Glaubensbekenntnis”).

26 It has been questioned in the literature whether this large set of items contributes to the accuracy of COMPAS; Dressel and Farid [14] claim that a simple linear model with two factors achieves the same accuracy as COMPAS.

27 Brennan and Dieterich [8] cite one study conducted in Broward County, which found that the use of COMPAS reduced jail time and saved money.

28 In the context of climate models, this corresponds to the adequacy-for-purpose view, cf. Parker [39].

29 See, e.g., Fazel [15, p. 18] on this issue in the context of recidivism risk.

30 In 2019, 6% of inmates in Swiss penitentiaries were female, and 74% were foreign citizens according to the Swiss Federal Statistical Office (“Strafvollzug: Mittlerer Insassenbestand nach Geschlecht, Nationalität und Alter”, status of Oct. 22, 2020); see also Maillard [36] for Swiss sentencing and reoffending statistics. In a 2013 evaluation of ROS, which prescribes the application of FOTRES in severe cases, females and foreign citizens were included without provision, cf. Schwarzenegger et al. [45, p. 21].

31 It would have been desirable to obtain a representative normative sample for FOTRES, as used in the adaptation of PCL-R, a Canadian instrument, to the context of Germany, cf. Hollerbach et al. [24].

32 “Different versions of COMPAS are customized for male and female offenders, youth offenders, internal prison classifications, and reentry versions.” [8, p. 50].

33 “The basic Core COMPAS risk models have been tested for predictive validity across diverse regions, on different criminal justice populations, gender and race groups, and also evaluated by independent research teams.” [8, p. 52].

34 See Jebeile et al. [27], Knüsel and Baumberger [30], Kuorikoski and Ylikoski [32], de Regt and Dieks [12] for proposals related to intelligibility. Intelligibility is closely related to the notion of *interpretability* from computer science; see Lipton [35], Rudin [43]. However, see also Krishnan [31], who argues that we should not focus on interpretability as a goal in and of itself.

35 In a discussion of decision 6B_424/2015 by the Swiss federal court, Vuille [57, p. 7] writes: “The Court [...] requires forensic reports, including those assessing an offender’s dangerousness, to be both transparent and traceable/understandable. Furthermore, this requirement is valid for the report as a whole, as well as for each of the instruments used by the expert to help him reach his conclusions.”

36 In the present paper, the focus is on “in principle” intelligibility. Of course, if an agent, say, a domain expert has intelligibility, this agent should be able to communicate their understanding to others in an adequate manner. The question of whether and how this works is of great importance, but it falls into the domain of science communication, which I will not explore here.

37 See Jebeile et al. [27] and references therein for more on this issue.

38 See Sullivan [51] for a philosophical discussion of understanding machine learning models in science.

39 See Rudin et al. [44] for discussion and criticism of the limited access to COMPAS due to its proprietary nature.

40 See www.fotres.ch, accessed on Feb. 11, 2021.

41 See [1, 14] for reasons in favor of simple, open source risk assessment instruments.

42 This issue is discussed in machine learning for more complicated models as adversarial attack, and more specifically as model stealing, where a private model is reconstructed based on publicly available information, cf. [55].

43 See Waggoner and Macmillen [58] for a discussion of the benefits of open source risk assessment instruments. I will not elaborate on the pros and cons of private v. public risk assessment instruments. Note that the intertwining of private and public sector with respect to FOTRES has been critically discussed in the press; see, e.g., Jan Jirát: “Chefarzt mit Nebenwirkungen”, WOZ, Nr. 45/2012.

44 “[The Swiss federal court] requires each instrument used in forensic expert reports to be traceable/understandable (*nachvollziehbar*). Under no circumstances can an instrument relying on a proprietary algorithm, such as FOTRES, comply with this requirement. [...][Defenants and their lawyers] must have access to the source code of any algorithm that is, even partially, influencing the judge’s decisions” [57, p. 30, italics in original].

45 See, e.g., Rudin et al. [44] for more discussion of this aspect of COMPAS.

46 Note that while the debate in computer science has focused on the fairness of machine learning models, we can ask about the fairness of any predictive model using concepts from machine learning.

47 See Berk et al. [6] for a useful overview of fairness in criminal risk assessment, and the first and last chapter of Singh et al. [50], where the importance of controlling the impact of risk assessment instruments on different social groups is emphasized.

48 See Barocas et al. [3] for an introduction to fair machine learning and for a recent discussion of these group fairness measures [59].

49 Via so-called impossibility theorems; see Barocas et al. [3] for discussion.

50 To give an example, to evaluate sufficiency with respect to the risk categories, the rate of reoffenders in each category has to be the same for the different groups. I thank an anonymous reviewer for pointing out this possibility.

51 “The FOTRES [...] provides an explanation regarding the level of risk for [the risk categories]. These explanations facilitate risk management decisions but are not estimates of risk [...] and thus do not permit the running of calibration statistics that compare the actual prevalence rates across the risk categories with the hypothesized categories” [42, p. 729].

52 See, e.g., Schwerzmann [46] for arguments in favor of abolishing structured risk assessment instruments altogether.
